# Supplementary material for: CYP7B1 as a Biomarker for Prostate Cancer Risk and Progression: Metabolic and Oncogenic Signatures (Diagnostic Immunohistochemistry Analysis by Tissue Microarray in Prostate Cancer Patients—Diamond Study)
Source: Int J Mol Sci. 2024 Apr 27;25(9):4762. doi: 10.3390/ijms25094762 (PMC11083792; doi:10.3390/ijms25094762)
Supplement: Supplementary file 1 [file ijms-25-04762-s001.zip › ijms-2960719-supplementary.pdf]

Suppl. Table S1. CYP7B1 expression according to IHC score in non-diabetic patients.

	CYP7B1		p-value
	N = 192	N = 106	
Age (years), median (Q1-Q3)	70.0 (64.0-75.0)	67.0 (63.0-71.0)	<0.01
PSA (ng/ml), median (Q1-Q3)	6.15 (3.70-9.20)	7.40 (5.70-11.40)	<0.01
Fasting glucose (mg/dl), median (Q1-Q3)	94.0 (85.0-101.0)	93.50 (86.0-101.0)	0.92
Total cholesterol (mg/dl), median (Q1-Q3)	191.0 (163.0-216.0)	192.0 (171.0-224.0)	0.37
Triglycerides (mg/dl), median (Q1-Q3)	107.0 (69.0-141.0)	103.0 (68.5-146.5)	0.84
Group, n (%)			<0.01
BPH	66 (34.48)	4 (3.77)	
PC	126 (65.62)	102 (96.23	
ISUP Gleason score, n (%)			0.32
1	42 (33.33)	26 (25.49)	
2	49 (38.89)	41 (40.20)	
3	26 (20.63)	22 (21.57)	
4	3 (2.38)	8 (7.84)	
5	6 (4.76)	5 (4.90)	
Pathological stage, n (%)			0.02
T2	93 (73.81)	69 (67.65)	
T3	26 (20.63)	16 (15.69)	
T4	7 (5.56)	17 (16.67)	
Classification risk of PC, n (%)			0.42
Low risk	49 (38.89)	33 (32.35)	
Intermediate risk	53 (42.06)	43 (42.16)	
High risk	24 (19.05)	26 (25.49)	
Ki-67 positive score, n (%)	24 (12.50)	17 (16.04)	0.39
AR positive score, n (%)	86 (44.79)	55 (51.89)	0.24
PSMA positive score, n (%)	61 (31.77)	56 (52.83)	<0.01
IR-α positive score, n (%)	107 (55.73)	101 (95.28)	<0.01
IR-β positive score, n (%)	4 (2.08)	16 (15.09)	<0.01
IGF-1R positive score, n (%)	28 (14.58)	22 (20.75)	0.17
ATPLy positive score, n (%)	72 (37.50)	71 (66.98)	<0.01
SRSF-1 positive score, n (%)	82 (41.71)	70 (66.04)	<0.01
CPT1-a positive score, n (%)	27 (14.06)	23 (21.70)	0.09
SCD-1 positive score, n (%)	28 (14.58)	20 (18.87)	0.33
SREBP1 positive score, n (%)	54 (28.12)	18 (16.98)	0.03
FAS positive score, n (%)	91 (47.40)	80 (75.47)	<0.01
ACC-1 positive score, n (%)	52 (27.08)	53 (50.0)	<0.01

IHC = Immunohistochemistry; BPH = Benign Prostatic Hyperplasia; PC = prostate cancer; Q1-Q3 = interquartile range; CYP7B1 = Cytochrome 7B1; AR = Androgenic receptor; PSA = Prostate-Specific antigen; IR=insulin receptor; IGF-1R = insulin-like growth factor-1 receptor; PSMA = prostate specific membrane antigen; SRSF-1 = Serine/arginine-rich splicing factor 1; FAS = fatty acid synthase; CPT-1a = Carnitine palmitoyltransferase 1a; SCD-1 = Stearoyl-CoA desaturase-1; SREBP-1 = Sterol regulatory element-binding protein-1; AC-1 = Acetyl-CoA Carboxylase-1.
